# Fibroblasts from idiopathic Parkinson’s disease exhibit deficiency of lysosomal glucocerebrosidase activity associated with reduced levels of the trafficking receptor LIMP2

**DOI:** 10.1186/s13041-020-00712-3

**Published:** 2021-01-19

**Authors:** Ria Thomas, Elizabeth B. Moloney, Zachary K. Macbain, Penelope J. Hallett, Ole Isacson

**Affiliations:** Neuroregeneration Research Institute, Harvard Medical School/McLean Hospital, Belmont, MA 02478 USA

**Keywords:** GBA, LIMP2, Idiopathic PD fibroblasts, Lysosomal dysfunction

## Abstract

Lysosomal dysfunction is a central pathway associated with Parkinson’s disease (PD) pathogenesis. Haploinsufficiency of the lysosomal hydrolase *GBA* (encoding glucocerebrosidase (GCase)) is one of the largest genetic risk factors for developing PD. Deficiencies in the activity of the GCase enzyme have been observed in human tissues from both genetic (harboring mutations in the *GBA* gene) and idiopathic forms of the disease. To understand the mechanisms behind the deficits of lysosomal GCase enzyme activity in idiopathic PD, this study utilized a large cohort of fibroblast cells from control subjects and PD patients with and without mutations in the *GBA* gene (N370S mutation) (control, n = 15; idiopathic PD, n = 31; PD with *GBA* N370S mutation, n = 6). The current data demonstrates that idiopathic PD fibroblasts devoid of any mutations in the *GBA* gene also exhibit reduction in lysosomal GCase activity, similar to those with the *GBA* N370S mutation. This reduced GCase enzyme activity in idiopathic PD cells was accompanied by decreased expression of the GBA trafficking receptor, LIMP2, and increased ER retention of the GBA protein in these cells. Importantly, in idiopathic PD fibroblasts LIMP2 protein levels correlated significantly with GCase activity, which was not the case in control subjects or in genetic PD *GBA* N370S cells. In conclusion, idiopathic PD fibroblasts have decreased GCase activity primarily driven by altered LIMP2-mediated transport of GBA to lysosome and the reduced GCase activity exhibited by  the genetic *GBA* N370S derived PD fibroblasts occurs through a different mechanism.

## Introduction

Parkinson’s disease (PD) is a multifactorial neurodegenerative disorder, and several cell biological pathways that contribute to PD etiology have been described, including mitochondrial dysfunction, oxidative stress, lysosomal dysfunction, lipid and lipid transport abnormalities, vesicular transport deficits, immune response activation, and protein aggregation pathways [1–3]. Recent studies highlight a critical role for lysosomal dysfunction in the etiology of genetic and sporadic forms of PD [4, 5]. Glucocerebrosidase (GCase), a lysosomal hydrolase encoded by the *GBA* gene, is responsible for the metabolism of the glycosphingolipid substrates glucosylceramide (GlcCer) and glucosylsphingosine (GlcSph). While homozygous mutations in this gene lead to the most prevalent lysosomal storage disorder Gaucher’s disease, heterozygous loss-of-function mutations in *GBA* are one of the most common genetic risk factors identified in PD [5–7]. Reduced GCase activity is observed in PD patient tissues and in in vitro cellular systems harboring mutations in *GBA* gene, as well as, in the brain, cerebrospinal fluid (CSF) and blood from sporadic PD patients with no *GBA* mutations [4, 8–17]. Aging is the most significant risk factor for developing PD and several pathological processes of the disease are phenocopied in normal aging. Interestingly, lysosomal GCase activity is reduced in normal aging in humans and rodents with parallel elevations of glycosphingolipids [4, 18]. Furthermore, overexpression of GBA in two rodent models of PD is protective against dopamine neuron degeneration and reduces alpha synucleinopathy [19]. Some studies have also postulated a potential interaction between GBA and alpha-synuclein whereby accumulation of the substrates GlcCer and GlcSph, dysfunction of autophagy and ubiquitin proteasome system observed in models with mutant *GBA* was associated with increased accumulation of alpha-synuclein, and increased level of alpha-synuclein result in reduced GCase activity [12, 20]

The activity and function of GBA is regulated by several accessory proteins including lysosomal integral membrane protein 2 (LIMP2) and progranulin (PGRN). Unlike other lysosomal enzymes that utilizes mannose 6 phosphate receptor for trafficking to lysosomes, GBA is transported from the endoplasmic reticulum (ER) through Golgi to lysosomes as part of a complex with the specific receptor LIMP2 (encoded by *SCARB2* gene) in association with adaptor proteins 1 and 3 [21–25]. Homozygous mice deficient for LIMP2 exhibit significantly reduced GCase activity in peripheral tissues with a concomitant increase in the level of its substrate GlcCer, and in several regions of the brain paralleled by increased alpha synuclein levels, neutral lipid load, and lysosomal dysfunction [24, 26]. Deficiency of LIMP2 in these animals caused mistargeting and extracellular secretion of the GBA protein leading to its increased expression and activity in serum [24]. Overexpression of functional LIMP2 in murine fibroblasts and human neuroglioma cells stably expressing alpha synuclein led to increased trafficking of the GBA protein from ER to post ER compartments, increased enzyme activity, and dose dependent reduction in alpha synuclein levels [26]. Genome-wide association studies (GWAS) studies in several cohorts have identified that two short nucleotide polymorphisms (SNPs), rs6825004 (located within the *SCARB2* gene) [27] and rs6812193 (located immediately upstream of *SCARB2* gene) [28–30], are significantly associated with sporadic PD. However, studies performed in populations with differing genetic backgrounds indicate that the PD risk generated by these polymorphisms may depend on such genetic contexts. [31–33]. Despite significant association with PD, clinical studies have also reported that LIMP2 transcript and protein levels, [34] and GCase activity [28] have not been shown to associate with these SNP genotypes.

PGRN, encoded by the *GRN* gene, is a secreted glycoprotein comprising seven and a half granulin motifs connected by short linker regions. Heterozygous mutations in *GRN* lead to the development of frontotemporal dementia (FTD) and homozygous mutations cause the lysosomal storage disorder, neuronal ceroid lipofuscinosis [35–37]. In addition to its role in regulating a multitude of functions including embryogenesis, tumorigenesis, inflammation and wound repair [38, 39], recent studies have identified its role in lysosomal function as a chaperone of several lysosomal enzymes such as GCase [40, 41], cathepsin D [42] and Hex A [43]. Two modes of regulation of GBA by PGRN have been identified so far. Within the cytosol, PGRN binds to GBA through the c-terminal granulin E domain and is required for the lysosomal localization of the GBA/LIMP2 complex [40]. Within the lysosomes, PGRN is required for the processing of prosaposin to saposinC, which in turn, acts a co-activator for GBA [44, 45]. Cortical neurons derived from FTD PGRN iPSCs display reduced processing of prosaposin to saposinC and reduced lysosomal GCase activity compared to isogenic controls [45].

Even though primarily a symptomatic neurological disease, PD is widely systemic with multiple cellular systems and organs affected [46, 47]. Even in the rare classic mendelian families with evident genetic etiology, the associated mutations and cellular phenotypes are present in all cells of the organism, so potentially there are also systemic interactions that lead to the neurological manifestations and neurodegenerative changes. The degeneration of specific cellular systems upon exposure to PD-associated environmental, aging and genetic stressors depends on increased intrinsic vulnerability of certain cell types to the disease-causing mechanisms, and even within the brain there is regional and cellular differences in susceptibility to PD pathology [48, 49]. Neurons are relatively more vulnerable than other cellular systems and organs, such as the 100-fold higher vulnerability of iPSC-derived neurons with *PINK1* and *LRRK2* mutations to mitochondrial stress compared to fibroblasts from the same patients [50]. In order to study systemic and cell biological mechanisms associated with PD, easily accessible, peripheral cell types such as fibroblasts and blood cells can be used as in vitro models [9, 51–55]. The current study utilized a large cohort of idiopathic and genetic (with *GBA* N370S mutation) PD patient-derived skin fibroblasts as in vitro cellular model to establish mechanisms leading to the deficiency of GCase in various forms of PD. Our findings identified that lysosomal GCase activity was reduced in idiopathic PD patient cells and was associated with decreased LIMP2 levels. These data confirm that GCase activity, and more broadly, lysosomal dysfunction is perturbed in idiopathic forms of PD, and suggest that reduced GBA function in idiopathic PD could be the result of a LIMP2-mediated trafficking deficit.

## Results

### Idiopathic PD patient fibroblasts display reduced basal GCase activity compared to healthy subject controls

**Fig. 1:**
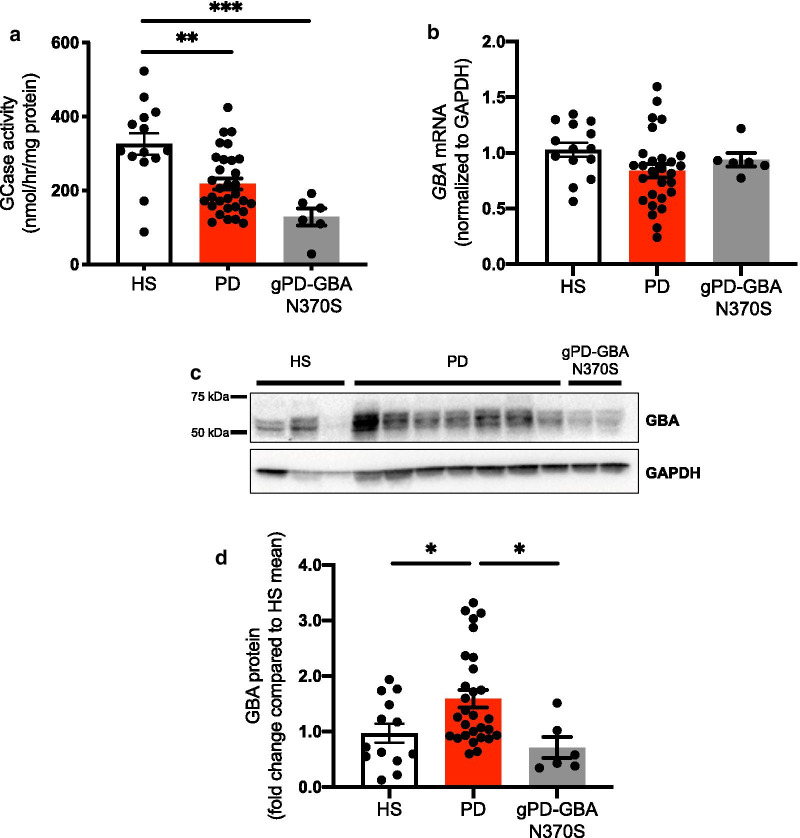
Idiopathic PD fibroblasts exhibit reduced basal lysosomal GCase activity. **a** Basal level of lysosomal GCase activity was measured in fibroblasts derived from idiopathic PD patients (PD), those harboring GBA N370S mutation (gPD-GBA N370S) and age-matched healthy subject controls (HS) using 4-MU glucopyranoside substrate (n = 14, HS; n = 31, PD; n = 6, gPD-GBA N370S; One-way ANOVA with Tukey’s multiple comparison test, F_(2,48)_ = 12.25, *p* < 0.0001). **b** *GBA* transcript (n = 14, HS; n = 28, PD; n = 6, gPD-GBA N370S) and **c**, **d** protein level (normalized to GAPDH) measured across HS, PD and gPD-GBA N370S group of cells (n = 13, HS; n = 29, PD; n = 6, gPD-GBA N370S; One-way ANOVA with Tukey’s multiple comparison test, F_(2,45)_ = 5.203, *p* = 0.0093). Data represented as mean ± SEM. * = *p* < 0.05; ** = *p* < 0.01; *** = *p* < 0.001

Our previous findings demonstrated that GCase activity progressively declines with age, and is decreased in PD patient substantia nigra compared to healthy patients [4]. To understand this further, in the current study a large cohort of fibroblasts derived from patients with idiopathic PD (PD), those harboring N370S mutation in the *GBA* gene (gPD-GBA N370S) and age-matched healthy subject controls (HS) were utilized to measure basal levels of lysosomal GCase activity. In addition to the expected reduction of GCase activity in gPD-GBA N370S cells (60.71%, *p* = 0.0001), a significant decrease in the enzyme activity was also observed in cells derived from idiopathic PD (33.27%, *p* = 0.0012) patients (Fig. 1a). All cell lines from the PD group of fibroblasts were sequenced for the entire *GBA* gene and were confirmed to be devoid of any nonsense and missense mutations in the coding region. To examine whether the reduction of GCase enzyme activity in PD cells was due to reduced transcript or protein level, qPCR and immunoblot assays were performed. While no difference in *GBA* transcript expression was observed between the three groups of cells (Fig. 1b), GBA protein level was significantly upregulated in PD cells compared to HS controls (p = 0.0461) and gPD-GBA N370S cells (*p* = 0.0331) (Fig. 1c, d). To exclude the possibility of altered lysosomal load contributing to the reduced lysosomal GCase activity in PD fibroblasts, an immunoblot assay was performed for LAMP1, and no change in its expression was observed between the PD-derived cells compared to HS controls (Additional file 1: Fig. S1A, B, Additional file 2). Furthermore, regression analysis performed between GCase activity and age of disease onset (PD, r = − 0.1259, *p* = 0.5487; gPD-GBA N370S, r = 0.7724, *p* = 0.1258) (Additional file 1: Fig. S2A, Additional file 2), and GCase activity and disease duration (PD, r = 0.3403, *p* = 0961; gPD-GBA N370S, r = − 0.7546, *p* = 0.1404) (Additional file 1: Fig. S2B, Additional file 2) across the idiopathic PD and gPD-GBA N370S cells showed no significant correlation between the variables in both groups of PD cells.

### GBA trafficking receptor LIMP2 is reduced and its levels correlate with GCase activity in idiopathic PD patient-derived fibroblast cells

**Fig. 2:**
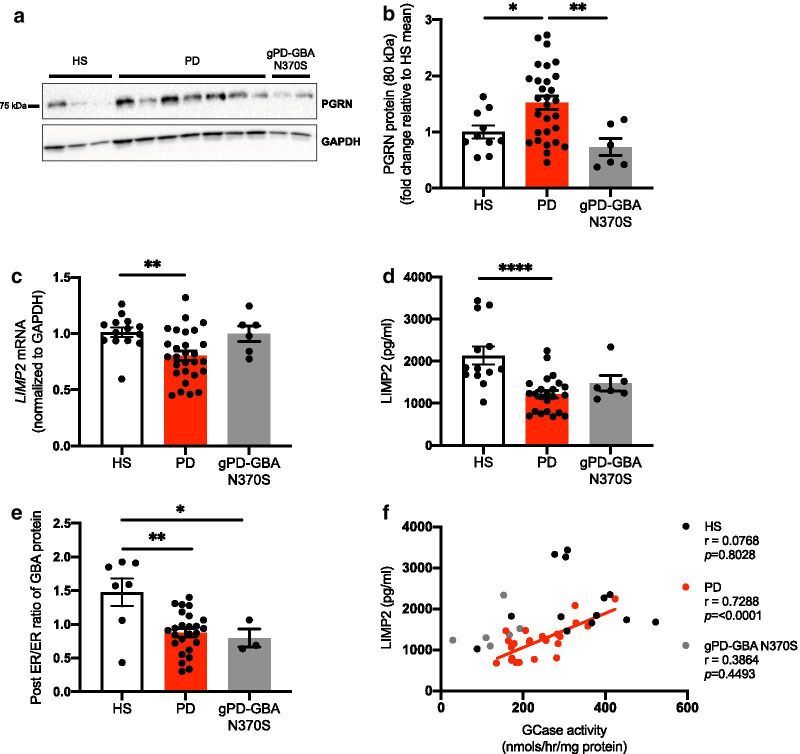
Idiopathic PD fibroblasts exhibit altered levels of PGRN, LIMP2 and localization of the GBA protein. **a** Representative image and **b** quantification of PGRN protein (normalized to GAPDH) in HS, PD and gPD-GBA N370S group of cells (n = 10, HS; n = 29, PD; n = 6, gPD-GBA N370S; One-way ANOVA with Tukey’s multiple comparison test, F_(2,42)_ = 6.577, *p* = 0.0033). **c** *LIMP2* transcript (n = 14, HS; n = 28, PD; n = 6, gPD-GBA N370S; One-way ANOVA with Tukey’s multiple comparison test, F_(2,45)_ = 6.147, *p* = 0.0044) and **d** ELISA-based LIMP2 protein expression across the three groups of cells (n = 13, HS; n = 23, PD; n = 6, gPD-GBA N370S; One-way ANOVA with Tukey’s multiple comparison test, F_(2,39)_ = 11.32, *p* = 0.0001). **e** Ratio of the post ER to ER fraction of GBA protein was measured in cell lysates using Endo-H and PNGase F digestion (n = 7, HS; n = 25, PD; n = 3, gPD-GBA N370S; One-way ANOVA with Tukey’s multiple comparison test, F_(2,32)_ = 8.084, *p* = 0.0014). **f** Correlation analysis between LIMP2 protein and GCase activity was performed and Pearson’s correlation coefficient was determined between the two variables across HS, PD and gPD-GBA N370S cells (n = 13, HS; n = 23, PD; n = 6, gPD-GBA N370S). Data represented as mean ± SEM. * = *p* < 0.05; ** = *p* < 0.01; **** = *p* < 0.0001

Activity of GBA is regulated by several accessory proteins and we analyzed two such proteins, PGRN and LIMP2, in detail. PGRN encoded by *GRN* gene has been reported to regulate GCase activity either directly by acting as a co-chaperone with Hsp70 [40], or indirectly by promoting the processing of prosaposin to saposin C [45]. While qPCR analysis for *GRN* transcripts showed a significant reduction in PD cells compared to controls (*p* = 0.01) (Additional file 1: Fig. S3A, Additional file 2), the protein levels were increased in this group of cells compared to HS (*p* = 0.0425) and gPD-GBA N370S cells (*p* = 0.0096) (Fig. 2A, B).

LIMP2 (also known as SCARB2) is the protein responsible for trafficking of GBA from ER to Golgi and finally to the lysosomes [24], and its expression was analyzed in this cohort of fibroblasts using qPCR and ELISA-based quantification assays. Interestingly, LIMP2 levels displayed a significant reduction at both the transcript (*p* = 0.0072) (Fig. 2c) and protein levels in PD (*p* < 0.0001) (Fig. 2d) cells compared to HS controls. Importantly, no change in this protein was observed in the gPD-GBA N370S cells (Fig. 2c, d). As the primary protein responsible for trafficking of GBA, its reduction in PD cells could lead to alteration in localization of the GBA protein. To examine this, Endo-H and PNGaseF digestion was performed in fibroblast lysates. GBA is heavily glycosylated and undergoes several carbohydrate modifications as it passes through the secretory pathway from ribosomes through ER to Golgi. The N-linked glycans present on GBA can be cleaved by the Endo-H enzyme (Endo-H-sensitive ER fraction). However, as the proteins pass through Golgi, these N-linked glycans are converted to complex oligosaccharides that are no longer responsive to the action of Endo-H enzyme (Endo-H-insensitive post ER fraction). The ratio of Endo-H-sensitive to -insensitive fractions of the GBA protein provides an indication of the localization of the protein between the ER and post ER compartments [9, 26]. Interestingly, both PD (*p* = 0.0013) and gPD-GBA N370S (*p* = 0.0266) cells displayed a reduction in post-ER/ER ratio compared to HS (Fig. 2e, Additional file 1: Fig. S3B, Additional file 2), suggesting a higher retention of the protein in the ER and thus an altered localization of the protein in both groups of cells. Importantly, regression analysis between LIMP2 and GCase activity levels across all cells showed that a significant correlation between the two variables was observed only in the case of idiopathic PD cells (r = 0.7288, *p* < 0.0001), and not in HS (r = 0.0768 *p* = 0.8028) or gPD-GBA N370S (r = 0.3864, *p* = 0.4493) cells (Fig. 2f).

Numerous GWAS have identified the presence of two SNPs, rs6825004 and rs6812193, to be associated with increased risk for PD in several study cohorts [27–30, 33]. Furthermore, both these SNPs are expression quantitative trace loci (eQTL) for SCARB2 in several tissues including cultured fibroblast cells [56]. To examine whether these nucleotide changes have a role in the reduced LIMP2 levels observed in PD fibroblasts, we sequenced a partial cohort of idiopathic PD (11 for rs6812193 and 14 for rs6825004 out of the total 31 PD lines) and gPD-GBA N370S (1 out of 6) cell lines for the two SNPs. Details of the distribution of genotype variants for rs6812193 and rs68225004 across the various cell lines sequenced in PD and gPD-GBA N370S groups are provided in a tabular form (Additional file 1: Fig. S4A, Additional file 2). Further analysis performed across the various SNP genotypes in the idiopathic PD group of cells showed that there was no significant difference in LIMP2 protein level nor lysosomal GCase activity between the cell lines with the various rs6812193 and rs6825004 polymorphisms (Additional file 1: Fig. S4B–E).

## Discussion

This study demonstrates that fibroblasts derived from idiopathic PD patients, devoid of mutations in the *GBA* gene, display reduced levels of lysosomal GCase activity, approaching that of cells with the *GBA* N370S mutation. In the fibroblasts from idiopathic PD patients, this GCase activity reduction was accompanied by a reduced protein expression of the GBA trafficking receptor, LIMP2, and by altered localization and increased ER retention of the GBA protein in these cells. Furthermore, regression analysis showed that LIMP2 expression levels correlated significantly with GCase activity levels only in idiopathic PD fibroblasts and not in  healthy subject or gPD-*GBA* N370S cells. Together, these results suggest that while reduced GCase activity is observed in fibroblasts derived from both idiopathic PD and a genetic form of PD with the *GBA* N370S mutation, reduced LIMP2 levels (both at transcript and protein level) are observed only in the case of idiopathic PD cells, indicative of different pathways leading to this deficit in these two PD cohorts. While the presence of the N370S mutation in the *GBA* gene is responsible for improper folding, increased retention of the protein in the ER, and increased lysosomal cholesterol accumulation, and thus reduced lysosomal GCase activity in gPD-GBA N370S group of cells [9], the reduced trafficking of GBA to lysosomes due to decreased LIMP2 levels could be responsible for the significantly reduced GCase enzymatic activity in the idiopathic PD cohort.

### Fibroblasts from idiopathic Parkinson’s disease patients exhibit reduced GCase activity levels

Numerous lines of research have identified lysosomal function to be a central pathway perturbed in PD [3, 57] and GWAS studies have identified heterozygous *GBA* variants and mutations to be present in 2–20% of PD patients from various cohorts [3, 5, 7, 58, 59]. In addition to a significantly large reduction of lysosomal GCase activity observed in tissues derived from PD patients harboring *GBA* mutations [8, 10, 15], intermediate deficits in this enzyme activity have also been observed in blood, CSF, and post-mortem brain tissue from sporadic PD patients [4, 8, 10, 13, 15]. Using a large cohort of fibroblasts (HS, n = 15; idiopathic PD, n = 31; gPD-GBA N370S, n = 6), this study demonstrates that, similar to deficiencies observed in the human post-mortem PD brain tissue, basal lysosomal GCase activity is highly reduced in gPD-GBA N370S cells (60.71%) and to an intermediary level in idiopathic PD cells (33.27%). Contrasting these results, three previous studies conducted in idiopathic PD fibroblasts concluded that these cells do not show differences in lysosomal GCase activity compared to controls [9, 60, 61]. However, the limited cohort of cell lines used in these studies (n = 2–5) might account for the observed differences between the results.

The reduced lysosomal GCase activity observed in fibroblasts derived from idiopathic PD patients in the current study was not due to changes in the corresponding transcript and protein expression. There was no reduction of mRNA expression and in fact, idiopathic PD fibroblasts exhibited a significant elevation of GBA protein compared to controls and gPD-GBA N370S cells, indicative of a possible cellular compensatory response. Reduced GCase activity in anterior cingulate cortex [13], cerebellum and substantia nigra [10] of sporadic PD post-mortem brain tissue was accompanied by reduced level of GBA protein. However, unlike patient-derived fibroblasts cells, post-mortem brain tissue represents an advanced stage of the disease, and it is plausible that mechanistic differences exist between these two tissue systems and cells under study. The GCase activity assay used in the current study specifically measures the activity of the lysosomal GBA enzyme and alterations in the overall lysosomal load within the cell can influence these results. No change was observed in the expression of the lysosomal marker LAMP1 between PD patient and control fibroblasts, suggesting that the decreased lysosomal GCase activity in idiopathic PD cells in the current study was not due to a general reduction of the lysosomal load. However, further experimental evidence using immunocytochemistry for lysosomal markers would serve to confirm this result. Furthermore, correlation analysis performed between lysosomal GCase activity levels and age of onset and disease duration excluded the influence of these factors in the reduced enzyme activity observed in idiopathic PD cells.

### Elevation of PGRN in idiopathic PD fibroblast cells

Investigation into one of the accessory proteins that regulate GBA function, PGRN, showed that while *GRN* transcript was downregulated, PGRN protein expression was significantly elevated in idiopathic PD cells. This discrepancy between transcript and protein level could suggest the presence of a post-translational modification leading to an increased half-life of the protein, and further experimentation would be required to confirm it. However, GCase activity is regulated by the PGRN protein (not the transcript) and its increase in PD cells, similar to that observed with GBA, could indicate a cellular compensation for reduced GCase activity in these cells. Along with HSP70, PGRN acts as a co-chaperone for GBA/LIMP2 complex and is required for its proper localization to the lysosome in vivo in PGRN KO  mice under ovalbumin-mediated inflammatory stress [40]. Overexpression of the complete PGRN protein or the c-terminal granulin E domain, which includes the binding site for GBA, abrogated the accumulation of GlcCer and increased GCase activity in fibroblasts isolated from Gaucher’s disease (GD) patients [40]. Furthermore, treatment of various GD mouse models (PGRN KO mice treated with ovalbumin and *GBA* D409V/- mice) with recombinant progranulin led to an increase in the appearance of lysosomal GCase, reduction of accumulated glycolipid substrates and the number and size of gaucher cells [62], indicating a potential therapeutic function for this protein in GD. However, despite the upregulation of PGRN in idiopathic PD fibroblasts in the current study, the lysosomal GCase activity was not increased. PGRN acts a co-chaperone of the GBA/LIMP2 complex and our results point to a reduction of LIMP2 in idiopathic PD cells. The decreased level of LIMP2 might serve as a limiting factor and could be the reason why lysosomal GCase activity was not rescued by the upregulation of PGRN in the idiopathic PD group of cells.

### Reduced expression of LIMP2 correlates significantly with reduced GCase activity in idiopathic PD cells

The significant downregulation of LIMP2, observed both at the transcript and protein level in idiopathic PD cells, but not in gPD-GBA N370S, suggests that the reduced lysosomal GCase activity in the former group of cells could be due to a defect in trafficking of the GBA protein. In line with this, idiopathic PD fibroblasts displayed an alteration in the localization of GBA protein, suggesting an increased retention of the protein in the ER compared to post-ER fraction. Additional experimentation using immunocytochemistry with GBA and an ER marker would serve to confirm this finding. We note that there is a trend for reduced LIMP2 protein level in gPD-GBA N370S cells, however, with the current sample size this did not reach statistical significance. Furthermore, regression analysis showed that a significant correlation of LIMP2 protein levels with GCase activity was observed only in the idiopathic PD group of cells, and not in HS and gPD-GBA N370S cells, further supporting the involvement of LIMP2 in the regulation of GBA enzyme activity exclusively in fibroblasts derived from idiopathic PD cohort. Changes in LIMP2 level can affect the pool of functional GCase enzyme entering the lysosome. A mutation in *LIMP2* (p.Glu471Gly) was identified to be a modifier of  GD and displayed inefficient lysosomal localization of GBA followed by accumulation of downstream GCase targets such as GlcSph and GlcCer [63]. Furthermore, LIMP2 deficient mice (LIMP2^−/−^) display diminished GCase activity (and protein) in the brain compared to WT littermate controls [24, 26]. These studies support our finding that reduced level of LIMP2 can indeed result in reduced lysosomal GCase activity. In a previous, unrelated study, staining for LIMP2 in PD human post-mortem brain tissue showed that the surviving dopaminergic neurons in the substantia nigra displayed elevated LIMP2 levels compared to controls [26]. However, differences between the two systems being studied here (fibroblasts and brain) and the stage of the disease they represent might account for the differences in the results.

Several GWAS studies identified that the two SNPs rs6825004 and rs6812193 exhibit a population-dependent association with PD [27–30] which prompted us to study the influence of these variances on LIMP2 level in our work. HS controls were not sequenced for these SNPs in our study, and hence, we cannot conclude on their association with PD based on our data. In line with the two previous reports [28, 34], our study provides further evidence that neither the LIMP2 level nor GCase activity are affected by the genotype at rs681193 or rs6825004 locus in PD fibroblast cells, and hence, does not explain the reduction of LIMP2 observed at the transcript and protein level in idiopathic PD group of cells compared to age matched controls. Closer analysis of these data show very marginal effects, in particular the eQTL inference is barely significant and does not indicate much change in expression levels. Further analysis, with increased sample size and consideration for the ethnicity of the study subjects would need to be performed to confirm any potential association of these polymorphisms with PD and the mechanisms by which they could, potentially, confer increased susceptibility to the disease.

## Conclusions

The current findings can help to define the cell biological mechanisms regulating GCase activity in idiopathic PD. Decreased levels of the GBA trafficking protein LIMP2, which was observed in idiopathic PD patient fibroblasts, would disrupt GBA trafficking from the ER/Golgi to lysosomes resulting in the observed reduction in lysosomal GCase activity. Alterations in lysosomal enzyme activities and subsequent lysosomal dysfunction can lead to deficits in cellular degradation pathways. In vulnerable cell types such as neurons, increased cellular glycosphingolipid load and lipid dyshomeostasis can precipitate abnormal protein, vesicular and neuroimmune interactions, eventually leading to their degeneration [1, 2, 64]. The current data also illustrate that cells peripheral to the brain, such as fibroblasts, can serve as platforms for experimental and therapeutic paradigms aimed at manipulation of cellular GCase levels. GBA related cell biological pathways seem to be dysregulated in the majority of PD cases. In addition to PD, mutations in GBA and reduced GCase activity are also observed in the related disorder, dementia with Lewy bodies (DLB) [65]. Interestingly, one of the *SCARB2* associated SNPs analyzed in this study (rs6812193) has also been identified to be a risk loci for DLB [66], and further studies to examine a LIMP2-associated influence on GCase activity in DLB would be of interest. Therapeutic modalities aimed at improving lysosomal function by increasing the activity of lysosomal enzymes, increasing lysosomal enzyme transport through LIMP2, or reducing glycolipid substrate accumulation, serve as attractive therapeutic targets and are currently under development.

## Methods

### Human dermal fibroblast lines

Healthy subject-derived fibroblasts (HS, n = 15), idiopathic PD patient (PD, n = 31), and mutant GBA PD patient fibroblast (gPD-GBA N370S, n = 6) lines were obtained from Coriell, and NINDS repositories (see Table 1 for overview). The skin samples used for generating the fibroblast cell lines were collected under Coriell and NINDS’s informed consent and deidentification of subjects. Biospecimens obtained from the NINDS Human Cell and Data Repository were not considered to be human subject research because conducting research with the samples does not involve an intervention or interaction with the individual and the samples do not contain identifiable private information. Idiopathic cells used in this study are defined as non-familial form of PD where cause of the disease is not known yet. Fibroblasts were maintained in culture as described previously [53], and cells were not used for experiments beyond passage 20. HS, PD and gPD-GBA N370S cells were age-matched and the average ages were 65, 67.2 and 68.6 years respectively. Apart from being sequenced for the *GBA* gene, a subset of the fibroblasts (indicated with *) were also sequenced and confirmed to be devoid of any mutations in the *LRRK2* gene as part of a previous study [53].

**Table 1:** Case information on the Parkinson’s disease and control fibroblast lines used in this study

Catalogue ID	Description	Sex	Age at biopsy
ND34769	Control	Female	68
ND34791	Control	Female	60
ND35046	Control	Male	60
ND36091	Control	Female	63
AG11743	Control	Female	76
AG06959	Control	Male	67
AG04061	Control	Male	66
AG13220	Control	Male	66
AG04355	Control	Male	67
AG11489	Control	Male	66
AG07141	Control	Male	66
AG05265	Control	Female	61
AG06010	Control	Female	62
AG06241	Control	Male	61
AG06281	Control	Male	67
AG20439	Idiopathic PD	Male	55
AG20445	Idiopathic PD	Male	60
ND30159*	Idiopathic PD	Female	76
ND35302*	Idiopathic PD	Male	69
ND35976*	Idiopathic PD	Male	63
ND39538*	Idiopathic PD	Female	72
ND39999*	Idiopathic PD	Male	63
ND34106*	Idiopathic PD	Male	65
ND29541*	Idiopathic PD	Male	65
ND39528*	Idiopathic PD	Female	67
ND39183	Idiopathic PD	Male	70
ND32462*	Idiopathic PD	Male	75
ND39955*	Idiopathic PD	Male	55
ND31508*	Idiopathic PD	Male	71
ND32157*	Idiopathic PD	Female	52
ND32697*	Idiopathic PD	Male	58
ND34265*	Idiopathic PD	Male	62
ND38528*	Idiopathic PD	Female	65
ND34854*	Idiopathic PD	Female	68
ND37609*	Idiopathic PD	Male	68
AG08395	Idiopathic PD	Female	85
ND29494	Idiopathic PD	Male	80
ND33424	Idiopathic PD	Male	57
ND38020	Idiopathic PD	Male	86
ND38865	Idiopathic PD	Male	51
ND38791	Idiopathic PD	Female	69
ND39450	Idiopathic PD	Female	72
ND39510	Idiopathic PD	Male	69
ND39957	Idiopathic PD	Female	70
ND41125	Idiopathic PD	Male	70
ND40260	Idiopathic PD	Male	78
ND29756	*GBA* N370S Het	Female	55
ND34982	*GBA* N370S Het	Female	82
ND34263	*GBA* N370S Hom	Male	65
ND35843	*GBA* N370S Hom	Male	61
ND31630	*GBA* N370S Het	Male	69
ND37180	*GBA* N370S Het	Male	80

### Glucocerebrosidase activity

Fibroblasts were plated onto tissue culture plates at 15,000 cells/cm^2^. They were supplemented with 0.1% DMSO in fibroblast culture medium the next day. Three days post-plating, the cells were harvested with lysis buffer (pH 7, 10 mM Tris, 0.1% Ipegal, 1 × Halt protease and phosphatase inhibitor cocktail with 0.5 M EDTA (Thermo fisher, #78440)) and mechanically homogenized with a sonicator (BioLogics Inc, Model 150 V). GCase activity was measured in cell lysates diluted three times in GCase-activity sample diluent (50 mM citric acid, 0.1 M sodium phosphate, and 2 mg/mL bovine serum albumin, pH 5). 10µL of diluted sample was added to 75µL of 5 mM 4-Mu-β-D-glucopyranoside (Sigma, #M3633) substrate prepared in GCase-activity substrate diluent (50 mM citric acid, 0.1 M sodium phosphate, 6 mg/mL sodium taurocholate (Sigma, #86339), 0.3% Tween20, pH 5). After incubation with the substrate for 60 min at 37 °C, the reaction was terminated using 200µL stop solution (333 mM glycine, 207 mM sodium carbonate, pH 10.7). Plates were read (Ex 360/Em 460) using a SPECTRAmax plate reader (Molecular Devices). Enzymatic activity of triplicate measurements of each sample was assessed from a 4-Mu standard curve (100–0.391 µM standard range prepared from a 1 mM 4-methylumbelliferyl sodium salt solution (Sigma, #M1508)) and normalized to total protein content in each sample.

### Sequencing of GBA gene and SNPs

DNA was extracted from fibroblast pellets using DNeasy blood and tissue kit (Qiagen, #69504). Primers between 18–22 bp and with a Tm of 52–62 °C were designed using the Primer3 software. DNA was amplified in a 25 ul reaction containing, 0.3 uM each PCR primers, 0.3 mM dNTP mix, 1X Kapa HiFi Fidelity Buffer with MgCl2 and 1U Kapa HiFi DNA Polymerase (Kapa Biosystems, #KR0368). The amplicon sizes for GBA gene, rs6812193 and rs68504 were 7766 bp, 554 bp and 552 bp respectively. PCR amplified products were pooled at an equimolar concentration and normalized to 0.2 ng/ul. Library construction of each amplicon pool to produce sequence ready indexed libraries was carried out using the Illumina Nextera XT DNA Sample Prep Kit as per manufacturer’s instructions. Quality control of the libraries were carried out by running them on a High Sensitivity DNA Tape on the Tapestation 2200 Instrument (Agilent Technologies) to measure library size (average size of libraries = 250 bp), and library concentrations were measured using the Quant-iT PicoGreen dsDNA BR Assay Kit (Life Technologies). Equimolar quantities of each uniquely indexed library were pooled and 10 pmol/L of the pooled libraries were subsequently run on the Illumina NextSeq 550 instrument to generate 150-bp paired-end sequencing reads. Generated sequencing reads were analyzed using a Burrows-Wheeler Aligner (BWA (mem), v0.7.17) for alignment, and the Genome Analysis Toolkit (GATK, (HaplotypeCaller) v4.0.3.0) unified Genotyper for variant calling (BWA and GATK are developed by the Broad Institute, Cambridge, MA). Genome amplification and sequencing was performed at the Partners HealthCare Personalized Medicine Translational Genomics Core (Cambridge, MA). The GBA sequencing carried out for this study were used in two previous publications [53, 67]. The primers used for the sequencing library construction are the following;

GBA, *Forward*: 5′ CGACTTTACAAACCTCCCTG 3′.

GBA, *Reverse*: 5′ CCAGATCCTATCTGTGCTGG 3′.

rs6812193, *Forward*: 5 CCCTAGGGGGAAATATGTGA 3′.

rs6812193, *Reverse*: 5′ TGTTCCTGCAGCTCCTTTTT 3′.

rs6825004, *Forward*: 5′ AAAGGACGTGTTTGTGTCCC 3′.

rs6825004, *Reverse*: 5′ AAAGCCATTCATTTTCAGGG 3′.

### Quantitative RT-PCR

RNA extraction was performed with the RNeasy Mini kit (Qiagen, #74104) and cDNA was synthesized using QuantiTect Reverse Tranascription kit (Qiagen, #205311) according to manufacturer’s instructions. qPCR reactions were performed using Power SYBR® Green PCR master mix (Thermo Fisher, #4367659) with 2 ng of cDNA and commercial primers (all Qiagen QuantiTect Primer Assays: *SCARB2*, #QT00041566; *GBA*, #QT00047551; *GRN*, Qiagen #QT01001686; and *GAPDH*, #QT00079247). qPCR reaction was run on a StepOnePlus real time PCR system (Applied Biosystems) and analysis was performed using the 2(-delta delta C(T)) method [68].

### Immunoblotting

Fibroblasts to be harvested for lysis were washed once with cold 1X PBS buffer followed by the addition of an appropriate amount of cold RIPA buffer (Thermo Fisher, #PI89900) supplemented with Halt protease along with phosphatase inhibitor cocktail and EDTA (Thermo fisher, #78440). The cells were scraped off the bottom of the culture dish and transferred to a microcentrifuge tube kept on ice. Cells were incubated on ice for 30 min after which they were sonicated (BioLogics Inc, Model 150 V) and spun down. Protein concentration of the supernatant was determined using BCA assay (Thermo fisher, #23225). Equal amount of protein was mixed with Pierce lane marker reducing sample buffer (Thermo fisher, #39000), boiled at 95 $^\circ{\rm C}$ for 5 min, loaded onto precast 4–20% gradient Criterion Tris–HCl protein gels (Bio-rad, #3450033) and was electrophoresed at 120 V for 2 h. The proteins were transferred to a PVDF membrane (Bio-rad, #1704157) using the Trans-blot turbo system (Bio-rad) at 25 V and 1.3 Amps for 15 min, followed by blocking of the membranes in blocking buffer comprising 1 × Tris-buffered saline (Bio-rad, #170-6435) with 0.1% Tween 20 (American Bioanalytical, #AB02038-01000) and 5% blotting grade blocker (Bio-rad, #170-6404). Membranes were then incubated overnight at 4 $^\circ{\rm C}$ (on a shaker) with the following primary antibodies diluted in blocking buffer: anti-GBA (Sigma, #G4171, 1:500), anti-PGRN (Sigma, #SAB4200310, 1:1000), anti LAMP1 (Abcam, #24170 1:1000) and anti-GAPDH (Sigma-Aldrich, #AB2302, 1:5000). The membranes were washed 4 times (10 min incubation on the shaker at room temperature) in TBST (1 × Tris-buffered saline (Bio-rad, #170-6435) with 0.1% Tween 20 (American Bioanalytical, #AB02038-01000) to remove excess unbound antibodies after which they were incubated in appropriate HRP conjugated secondary antibodies (Jackson Immunoresearch, #103-035-155, 1:5000; Bio-rad, #1706515, 1:5000) diluted in blocking buffer, for 1 h at room temperature (on the shaker). Following another 4 washes with TBST (10 min incubations on the shaker at room temperature), the membranes were developed using Advansta WesternBright Sirius chemiluminescent substrate (Advansta, K-12043-D20) or SuperSignal West Pico Plus chemiluminescent substrate (Thermo fisher, #34579), and imaged using Chemidoc XRS with Image Lab software. Densitometry analysis was performed using ImageJ software and all protein bands were normalized to GAPDH.

### ELISA-based measurement of LIMP2 protein levels

Equal concentration of cell lysates (RIPA lysates collected as mentioned above) were used to perform ELISA assay (RayBiotech, #ELH-LIMP-II) according to the manufacturer’s instructions. Samples and standards were run in triplicates and the final colorimetric readout was performed using a SPECTRAmax plate reader (Molecular Devices).

### Endo H and PNGase F treatment of cell lysates

Endo H (New England BioLabs, #P0702L) and PNGase F (New England BioLabs, #P0704L) was used according to manufacturer’s instructions. 20ug of cell lysates (RIPA lysates collected as mentioned above) was digested using 250 units of Endo H and 1000 units of PNGase F enzymes after which they were used for immunoblotting. A condition that included all steps of the digestion without the inclusion of the enzymes was included as a negative control.

### Statistical analysis

Statistical data analysis was performed in GraphPad Prism software version 8.4.2. All data are expressed as arithmetic mean $\pm$ SEM. Unpaired two-tailed student’s t-test or One-way ANOVA followed by post hoc testing was used as appropriate and the test used for each analysis is mentioned in the figure legend. In all cases, outliers identified using the iterative Grubb's function in GraphPad Prism, with alpha set at 0.05, were removed from subsequent analyses. *P* value < 0.05 was considered significant for all analyses.

## Supplementary Information

**Additional file 1: Figure S1.** Lysosomal load was not altered in PD patient-derived cells. (A) Representative image and (B) quantification of LAMP1 level (normalized to GAPDH) in whole cell lysates from HS, PD and gPD-GBA N370S cells (n = 13, HS; n = 28, PD; n = 6, gPD-GBA N370S; One-way ANOVA with Tukey’s multiple comparison test). Data represented as mean ± SEM. **Figure S2**: GCase activity does not correlate with the age of onset or disease duration in PD and gPD-GBA N370S group of cells. Correlation analysis between (A) GCase activity and age of onset (years) and, (B) GCase activity and disease duration (years) was performed in PD and gPD-GBA N370S group of cells and Pearson’s correlation coefficient was determined between the two variables (n = 25, PD; n = 5, gPD-GBA N370S). **Figure S3**: *GRN* transcript was reduced in idiopathic PD cells. (A) GRN transcript levels were measured across HS, PD and gPD-GBA N370S cells (n = 13, HS; n = 24, PD; n = 6, gPD-GBA N370S; One-way ANOVA with Tukey’s multiple comparison test, F_(2,40)_ = 4.772, *p* = 0.0138) using qPCR. (B) Representative image of immunoblot performed using Endo-H and PNGaseF digested lysates from HS, PD and gPD-GBA N370S cells for GBA protein (Quantification in Fig. 2E). Data represented as mean ± SEM. * = *p* < 0.05. **Figure S4**: LIMP2 and GCase activity levels do not correlate with rs6812193 or rs6825004 genotypes in idiopathic PD cells. (A) Table depicting the distribution of various genotypes at rs6812193 and rs6825004 locus across the cells from PD and gPD-GBA N370S group of cells. (B, C) LIMP2 levels and (D, E) GCase activity levels between PD cells with various genotypes for rs6812193 and rs6825004 SNPs. Data represented as mean ± SEM. **Figure S5**: Uncropped immunoblots used in the manuscript. Uncropped images of blots from (A) Fig. 1C, (B) Fig. 2A, (C) Supplementary Fig. 1A and (D) Supplementary Fig. 3B.

**Additional file 2.** Information on processed data used to generate all figures in this study.

## Data Availability

All results and methods utilized in this study are mentioned in the article and the supplementary information files. Individual data values for each of the analyses can be obtained from the corresponding authors upon reasonable request.

## Abbreviations

PD: Parkinson’s disease
GCase: Glucocerebrosidase
GlcCer: Glucosylceramide
GlcSph: Glucosylsphingosine
LIMP2: Lysosomal integral protein 2
PGRN: Progranulin
ER: Endoplasmic reticulum
GWAS: Genome wide association studies
SNPs: Short nucleotide polymorphisms
CSF: Cerebrospinal fluid
GD: Gaucher’s disease
DLB: Dementia with lewy bodies
