# Supplementary material for: Fibroblasts from idiopathic Parkinson’s disease exhibit deficiency of lysosomal glucocerebrosidase activity associated with reduced levels of the trafficking receptor LIMP2
Source: Mol Brain. 2021 Jan 19;14:16. doi: 10.1186/s13041-020-00712-3 (PMC7816505; doi:10.1186/s13041-020-00712-3)
Supplement: Supplementary file 1 — Additional file 1: Figure S1. Lysosomal load was not altered in PD patient-derived cells. (A) Representative image and (B) quantification of LAMP1 level (normalized to GAPDH) in whole cell lysates from HS, PD and gPD-GBA N370S cells (n = 13, HS; n = 28, PD; n = 6, gPD-GBA N370S; One-way ANOVA with Tukey’s multiple comparison test). Data represented as mean ± SEM. Figure S2: GCase activity does not correlate with the age of onset or disease duration in PD and gPD-GBA N370S group of cells. Correlation analysis between (A) GCase activity and age of onset (years) and, (B) GCase activity and disease duration (years) was performed in PD and gPD-GBA N370S group of cells and Pearson’s correlation coefficient was determined between the two variables (n = 25, PD; n = 5, gPD-GBA N370S). Figure S3: GRN transcript was reduced in idiopathic PD cells. (A) GRN transcript levels were measured across HS, PD and gPD-GBA N370S cells (n = 13, HS; n = 24, PD; n = 6, gPD-GBA N370S; One-way ANOVA with Tukey’s multiple comparison test, F(2,40) = 4.772, p = 0.0138) using qPCR. (B) Representative image of immunoblot performed using Endo-H and PNGaseF digested lysates from HS, PD and gPD-GBA N370S cells for GBA protein (Quantification in Fig. 2E). Data represented as mean ± SEM. * = p < 0.05. Figure S4: LIMP2 and GCase activity levels do not correlate with rs6812193 or rs6825004 genotypes in idiopathic PD cells. (A) Table depicting the distribution of various genotypes at rs6812193 and rs6825004 locus across the cells from PD and gPD-GBA N370S group of cells. (B, C) LIMP2 levels and (D, E) GCase activity levels between PD cells with various genotypes for rs6812193 and rs6825004 SNPs. Data represented as mean ± SEM. Figure S5: Uncropped immunoblots used in the manuscript. Uncropped images of blots from (A) Fig. 1C, (B) Fig. 2A, (C) Supplementary Fig. 1A and (D) Supplementary Fig. 3B. [file 13041_2020_712_MOESM1_ESM.docx]

**Additional Figure 1**

**Additional Figure 2**

**
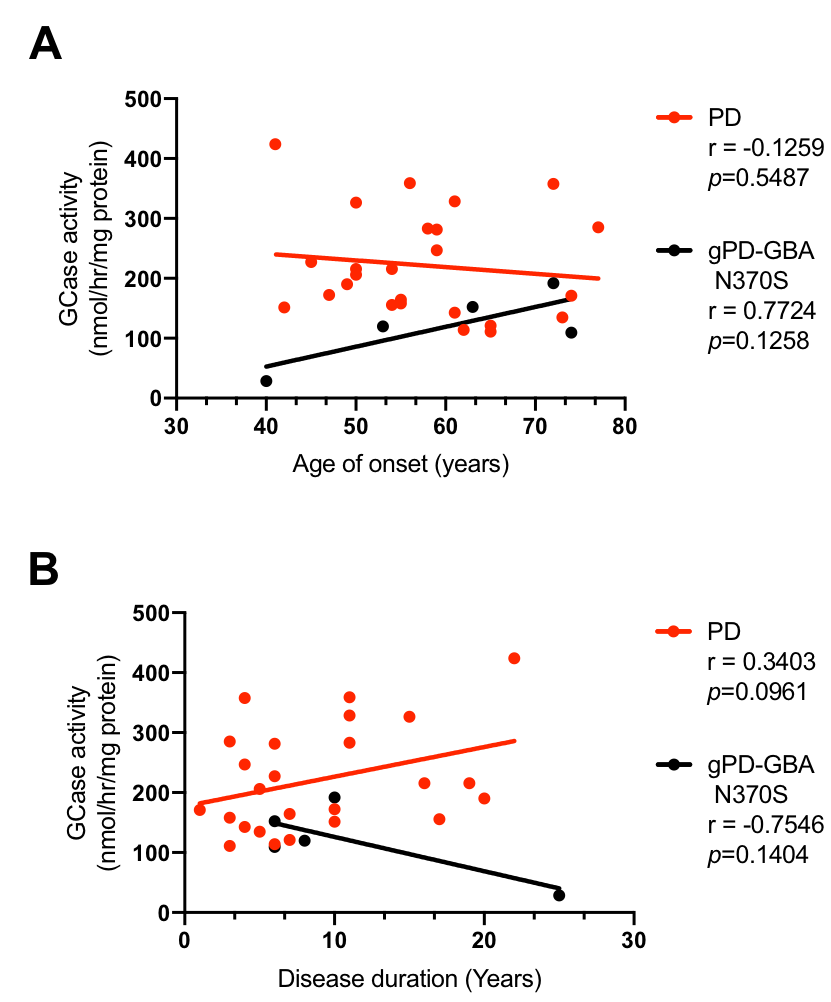
**

**Additional Figure 3**

**Additional Figure 4**

**
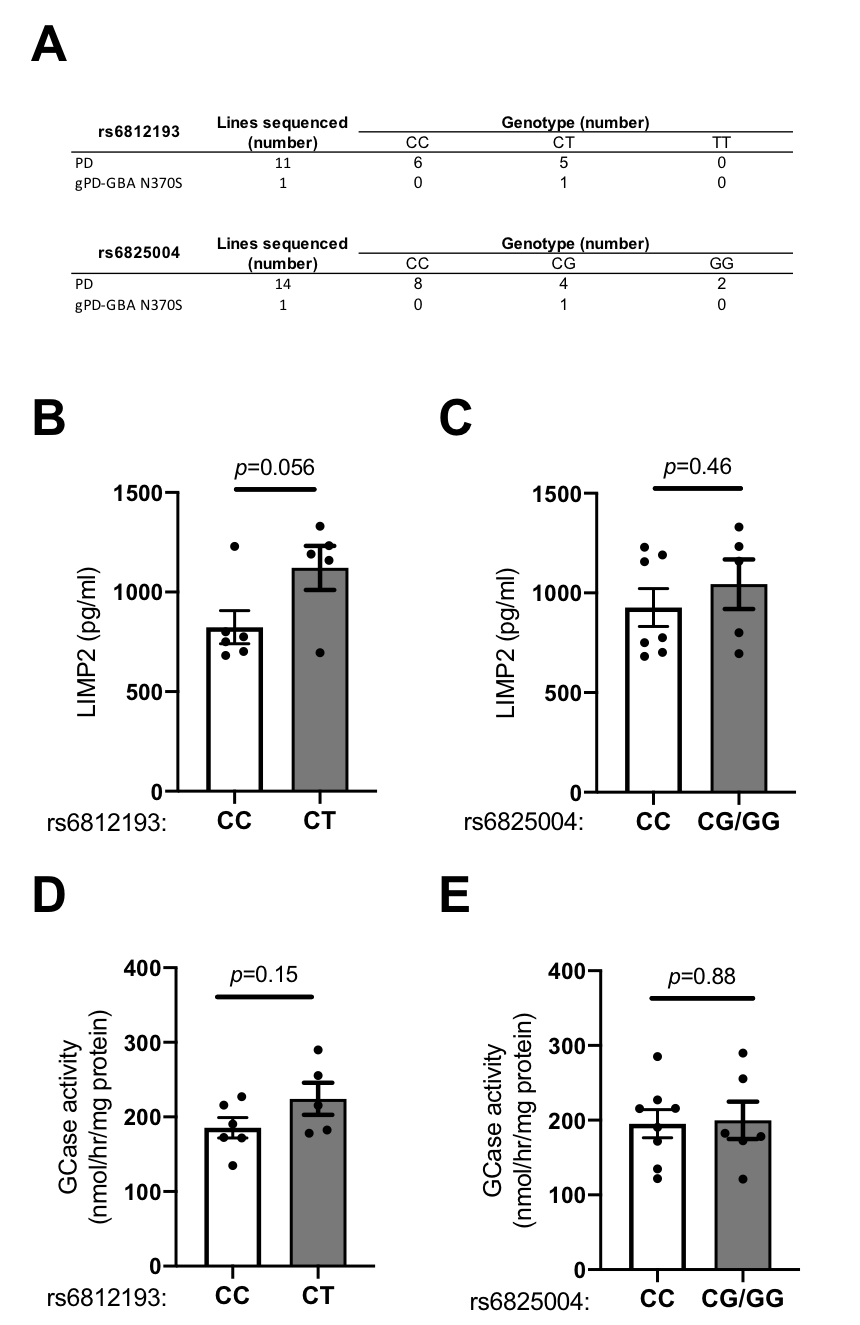
**

**Additional Figure 5**

**
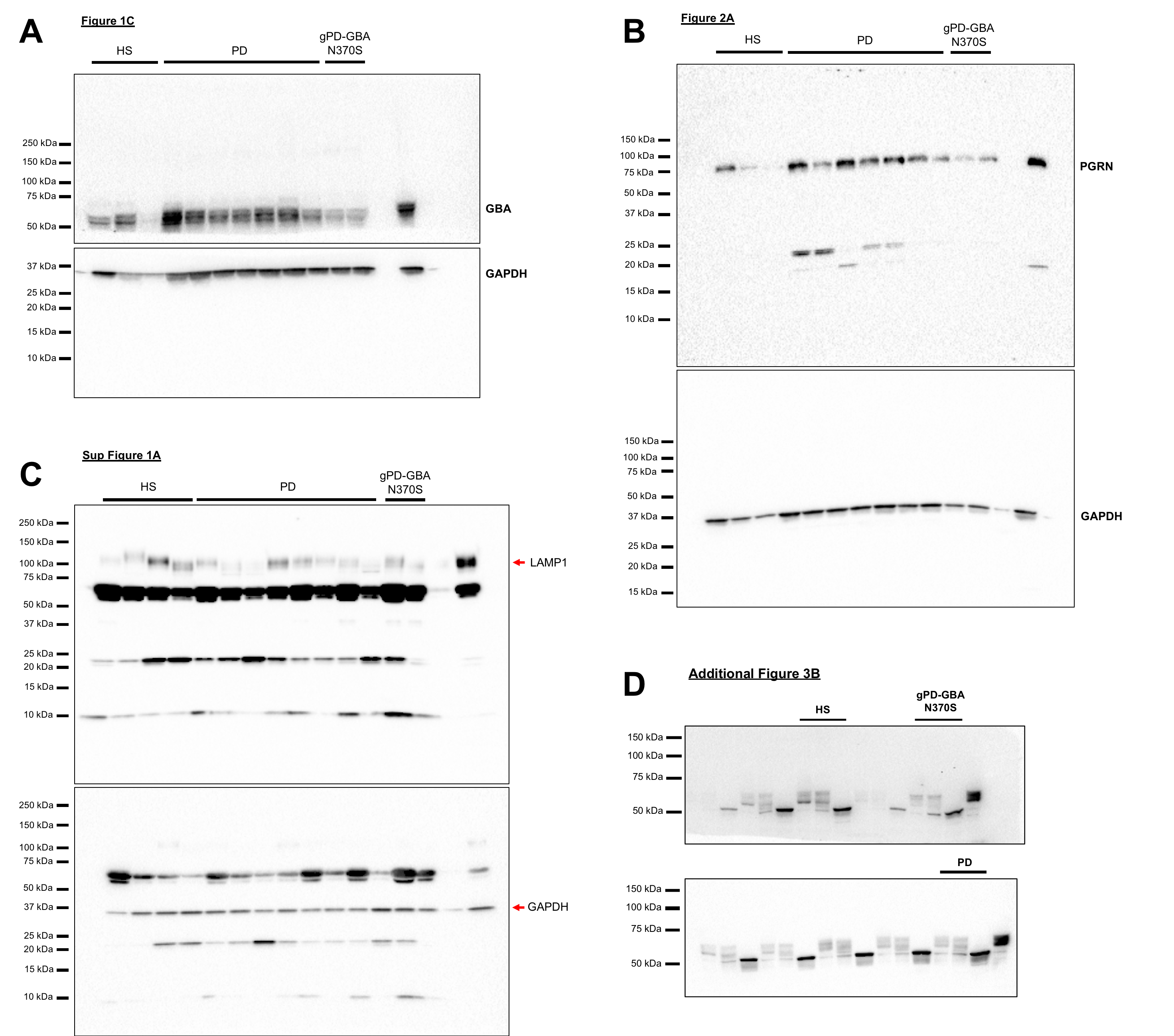
**
